# Focusing Narrowly or Broadly Attention When Judging Categorical and Coordinate Spatial Relations: A MEG Study

**DOI:** 10.1371/journal.pone.0083434

**Published:** 2013-12-26

**Authors:** Raffaella Franciotti, Stefania D’Ascenzo, Alberto Di Domenico, Marco Onofrj, Luca Tommasi, Bruno Laeng

**Affiliations:** 1 Department of Neuroscience and Imaging, G. d’Annunzio University, Chieti, Italy; 2 ITAB, “G. d’Annunzio” University Foundation, Chieti, Italy; 3 Department of Psychology, Humanities and Territory, G. d’Annunzio University, Chieti, Italy; 4 Department of Psychology, University of Oslo, Oslo, Norway; Centre de Neuroscience Cognitive, France

## Abstract

We measured activity in the dorsal system of the human cortex with magnetoencephalography (MEG) during a matching-to-sample plus cueing paradigm, where participants judged the occurrence of changes in either categorical or coordinate spatial relations (e.g., exchanges of left versus right positions or changes in the relative distances) between images of pairs of animals. The attention window was primed in each trial to be either small or large by using cues that immediately preceded the matching image. In this manner, we could assess the modulatory effects of the scope of attention on the activity of the dorsal system of the human cortex during spatial relations processing. The MEG measurements revealed that large spatial cues yielded greater activations and longer peak latencies in the right inferior parietal lobe for coordinate trials, whereas small cues yielded greater activations and longer peak latencies in the left inferior parietal lobe for categorical trials. The activity in the superior parietal lobe, middle frontal gyrus, and visual cortex, was also modulated by the size of the spatial cues and by the type of spatial relation change. The present results support the theory that the lateralization of each kind of spatial processing hinges on differences in the sizes of regions of space attended to by the two hemispheres. In addition, the present findings are inconsistent with the idea of a right-hemispheric dominance for all kinds of challenging spatial tasks, since response times and accuracy rates showed that the categorical spatial relation task was more difficult than the coordinate task and the cortical activations were overall greater in the left hemisphere than in the right hemisphere.

## Introduction

A fundamental goal of representations of space surrounding the body is to allow navigation and control of action within the physical environment. For such a goal, the brain supports a representation of space that is extended and continuous along quantitative coordinates. As humans, however, we do not simply “act” in space, but we also “know” it, and “talk” about it, thus our cognition of space can be also qualitative or “categorical” [1].

According to a recent theoretical approach of cognitive neuroscience, multiple spatial functions are differentially distributed across the “dorsal” system of both hemispheres in humans. Specifically, in contrast to the dominant idea that the right hemisphere is the “seat” of all possible spatial judgments, reasoning, and thought, Kosslyn [2], [3], proposed a neural (and computational) architecture [4], where separate subsystems process either quantitative aspects of spatial cognition (e.g., ‘how far’ or ‘how large’ is something) or qualitative aspects (e.g., whether something ‘is to the left’ or ‘is above’), so that at least two different properties can be extracted from specifications of location.

Separate spatial subsystems may have originally evolved to assist the solution of different spatial and object recognition problems [5]–[8] by representing in parallel the same spatial layout in at least two separate manners, a prevalently right-hemispheric mode that assesses spatial “analog” or “coordinate” spatial relations (e.g., the distance between two objects) and a prevalently left-hemispheric mode that assesses “digital” or “categorical” spatial relations (e.g., whether two objects are attached to one another, or one is above or below the other). By computing separately the two types of spatial relations (instead of taking the quantitative representation and making it coarser by grouping the finer locations) the brain could achieve a more efficient representation of space, where both properties can be attended simultaneously.

In fact, the clinical literature clearly indicates that not every spatial function depends on right-hemispheric function: damage to the left hemisphere results in problems with spatial judgments like deciding what is to the left versus what is to the right (i.e., Gerstmann’s syndrome; [9]) or in deficits in analysing spatial arrays of objects (i.e., “constructional apraxia” [10]). Neuroimaging studies [11]–[13] have recently shown that categorical spatial tasks engage regions of the left parietal lobe whereas coordinate spatial tasks engage the same areas in the right hemisphere. Moreover, areas of the left and right prefrontal cortex receiving direct input from ipsilateral parietal areas show activity when categorical or coordinate spatial information is held in memory [14]–[16]. Artificial neural network simulations on different types of spatial relations have shown that a more efficient processing can be achieved by “split” networks than unitary networks [4], [17]. These studies have also shown that, when trained to make either digital or analog spatial judgments, the networks encode more effectively each spatial relation if the input is based on units with relatively small, non-overlapping receptive fields, as opposed to units with relatively large, overlapping receptive fields [4]. Overlap of location detectors would then promote the representation of distance, based on a “coarse coding” strategy [18]–[21], whereas minimal or absent overlap between the units can benefit the representation of digital or categorical spatial relations, by effectively parsing space. Consistent with the above computational account, Laeng and colleagues [22] have shown that, manipulating the scope of the attention window can modulate the ability to represent each type of spatial relation [23], [24]. Specifically, they [22] found an interactive effect of spatial relation and visual field, so that response times (RTs) were faster to presentation of categorical changes in the right visual field (RVF) than left visual field (LVF), which indicates a specialization of the contralateral left hemisphere (LH) for categorical spatial relations. Analogously, RTs were faster to presentation of coordinate changes of the left visual field (LVF) than right visual field (RVF), which indicates a specialization of the contralateral right hemisphere (RH) for coordinate spatial relations.

A recent fMRI study [25] supports the idea that the cerebral specializations for categorical and coordinate spatial processing hinge on differences in the size of regions of space attended by the two hemispheres. In their study, participants memorized the position of dots and indicated whether a subsequent dot position differed categorically (opposite quadrant of the cross) or coordinately (same distance from the centre of the screen). The BOLD responses across the retinotopic maps of V1, V2, and V3 indicated that the spatial distribution of cortical activity was different in each task during the interval between the presentation of the sample and that of the match. Remarkably, a local focus of activity limited to one retinotopic quadrant was found during categorical processing, whereas activity was spread over several quadrants for coordinate processing, particularly in area V3. Such a difference in the spread of activation within visual areas during each spatial task was interpreted by van der Ham and colleagues [25] as evidence that engaging in different kinds of spatial judgments would result in spontaneous adjustments of the attention window (from local to global for categorical vs. coordinate relations, respectively). As van der Ham and colleagues pointed out, these differences in the extension of BOLD responses over the retinotopic maps are highly consistent with the hypothesis put forward by Laeng et al. [22], as well as Borst and Kosslyn [23], that attention should be distributed differently for an optimal processing of the type of spatial relation. However, in van der Ham et al.s’ study [25], hemispheric differences were not explored in relation to the spatial task and the BOLD response was analyzed during the interval between the presentation of the sample and that of the match, so as to mainly reflect working memory mechanisms occurring before the actual spatial comparison between the sample and the match was made.

In the present study, we relied on one direct and effective manner to test the hypothesis that the spatial area monitored by attention is relevant to the cognitive judgment, which consists in manipulating the scope of attention between the presentation of the sample and the match stimuli. That is, the focus of attention can be either spatially narrowed or distributed using cues of different size that briefly precede the appearance of a target [26]–[30], so that each cue can prompt the observers to adjust the aperture of the attention window to the perceived extent of each cue [31], [32]. Specifically, we predicted that narrowing attention to encompass an area that includes only one of the objects would benefit categorical spatial relations. In contrast, we predicted that spreading the attention window to encompass an area that includes two objects would promote coordinate spatial relations. These predictions derive from the hypothesis that attending a small area should exclude all location detectors that monitor areas of space larger than the region attended or that “overflow” the size of the attention window [22]. In other words, small attention cues that narrow attention to a scale that is smaller than the space subtended by both objects should benefit categorical judgments because attention would “divide” into optimally scaled regions; at the same time such a process will not benefit coordinate judgments as precise localization [33]. In contrast, larger attention cues should allow attending an area as large as that containing both objects, promoting the selection of units with large receptive fields, thus resulting in an increased overlap of spatial detectors. Therefore, in contrast to the effect of small attentional cues, the large cues should specifically promote the encoding of the quantitative or coordinate spatial properties.

In the present experiment, participants were not specifically instructed to pay attention to the cues, instead we assumed, on the basis of the previous studies by Laeng et al. [22], and Okubo et al. [34], that the differently sized cues would transiently capture the attention window and lead to a subsequent adjustment of the scope of attention in about 100 ms [35], [36]. In general, the facilitating effects of exogenous attention last no more than about 200 ms [27], [37] and cues of different sizes fail to have a differential effect on performance when they precede the target at very short intervals e.g., 100 ms [38]. However, observers can sustain attention in an endogenous manner to the cued aperture [39]–[42] and this will lead, according to the present hypotheses, to differential effects on performance.

According to previous neuropsychological and neuroimaging studies that have shed light on the functional neuroanatomy of spatial judgments of categorical versus coordinate type [6], [14], [15], [43], we hypothesize that “narrow” attention cues for categorical spatial transformation engage areas within the dorsal system (i.e., inferior and superior parietal lobe as well as prefrontal cortex) of the left hemisphere. In contrast, we expect that when the attention window is large, coordinate spatial transformations are better engaged within the same areas of the dorsal system, although in the other, right hemisphere.

We used magnetoencephalography (MEG) to observe differences in hemispheric activity related to the effect of large and small attentional cues on spatial relations. In the present experiment we used the same stimuli and spatial attention cueing procedure of Laeng and colleagues’ study [22], [34]. Differently from the experimental tasks used in previous neuroimaging studies, in which the coordinate judgment task was more difficult than the categorical task (i.e., yielded higher error rates and longer response times), in such a paradigm it is the categorical task that is clearly the more difficult between the two. Because RTs and accuracy index the difficulty of a task, we predict that a categorical change, being more difficult, will engage the left hemisphere more than the right and in particular when the stimuli are presented in the right hemifield [22]. Importantly, the present paradigm allows assessing the alternative idea [44] that difficult spatial judgments engage a “dominant” (for spatial perception and memory) right hemisphere. If the right hemisphere is truly dominant for all kinds of spatial judgements, then the present categorical task, being more difficult in our paradigm, should result in greater activity in the right than left hemisphere. In contrast, according to the present theory of complementary lateralized spatial representations [6], [22], increased difficulty for one type of spatial relation judgment will result in greater activity in the hemisphere specialized for that specific spatial task (i.e., the left hemisphere). That is, we assume that activity in dedicated neural networks is proportional to the extent and intensity of processing of neurons forming such networks [45]. Finally, one should note that the predictions that the level of activity within a hemisphere for each spatial task will be modulated by changes in the scope of the focus of attention cannot be derived on the basis of either task difficulty or a generic right-hemispheric specialization for visual attentional control.

## Materials and Methods

### Subjects

Twenty-two participants (12 females, mean age 26±3, ranging from 21 to 33 years) were recruited for the study. All participants were right-handers and had visual acuity of 20/20 or corrected to normal. None had a history of ophthalmic or neurological abnormalities.

### Ethics Statement

All subjects signed an informed written consent before recording; the experimental procedures were carried out according to the Declaration of Helsinki and they were previously approved by the local Institutional Ethics Committee (at the University of Chieti-Pescara, Italy).

Data will be made freely available upon request.

### Stimuli

**Figure 1:**
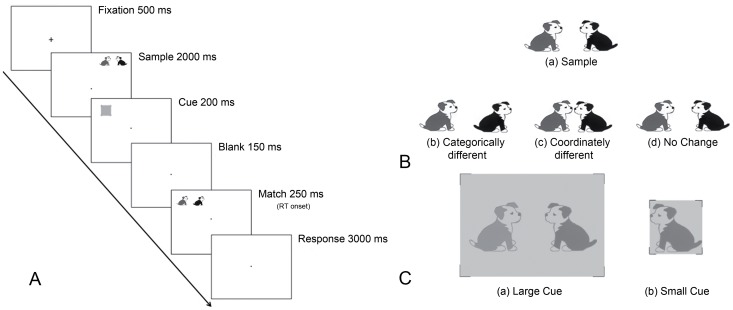
Experimental paradigm. A: Example of an experimental valid trial of the categorically (CAT) different condition with its timing; B: Example of sample stimulus (a) and matching stimulus when it is categorically different (b, CAT), coordinately different (c, COO) and identical (d, NoCh) to the sample stimulus; C: Attention window with large (a) and small cue (b).

All of the experimental trials consisted in comparing a sample stimulus and a matching stimulus, both types of stimuli including images of a pair of animals (see Figure 1 for examples of stimuli used in the task). The stimuli were coloured drawings of animals (e.g., dog, cat, bird) already used in previous studies [6], [10]. In the sample stimulus, the two animals were either facing one another or facing away from each other and they were separated in space by a distance subtending an angle of 1.6 degrees. Three possible pairing conditions of the same animals were used to create matching stimuli: coordinately different, categorically different, and with no change in spatial relations (Figure 1 B). For the coordinately different condition (COO), the distance between the two animals decreased (subtending an angle of 0.4 degrees) in comparison to the sample stimulus while their relative orientation remained unchanged. For the categorically different condition (CAT), the facing direction of one of the animals was reversed in comparison to the sample while the distance between animals remained unchanged. For the ‘no change’ condition (NoCh), the matching stimulus was exactly the same as the sample stimulus. The size of each animal in the pair subtended approximately 2 degrees of visual angle on the screen.

Gray squares, slightly darker than the white background of the screen, were used as cues to shift attention (Figure 1 C). The square cues came in two sizes, referred to as either ‘large’ or ‘small’. The large cue occupied a region of the screen that tightly included both animals of the stimulus pair, subtending an angle of 6 degrees, whereas the small cue occupied a region of the screen corresponding to the figure of a single animal, subtending an angle of 2 degrees.

### Procedure

In each trial, the sample stimulus was presented in one of the four possible quadrants: top left, top right, bottom left, bottom right. Both the sample and the matching stimulus were presented peripherally (6°), but at a different location within a trial.

In the cue-valid trials, the cue location was superimposed on the to-be-presented location of the matching stimulus. In the cue-invalid condition, the cue was presented at one of the two stimulus positions, where the matching and the sample stimuli were not shown in the same trial. Both cue-valid and cue-invalid trials were presented. Figure 1 illustrates the stimulus sequence in an experimental trial of the CAT condition. At the beginning of each trial, a fixation cross was presented in the centre of the screen for 500 ms. Next, a sample stimulus appeared for 2000 ms, followed by the cue (for 200 ms). Immediately after the cue, the screen turned blank for 150 ms, followed by a matching stimulus presented for 250 ms.

Each participant was comfortably seated in a dark room approximately 2 m away from the display with her/his head firmly in place, and placed the index fingers of both hands on the two-key response console. Response hands were counterbalanced across participants. They were instructed to keep the head still inside the helmet-shaped MEG system and to maintain their gaze at the fixation point, indicated by a black dot in the centre of the screen.

Participants were trained to respond as fast and accurately as possible after the presentation of the matching stimulus indicating whether it was the same as the sample stimulus or different from it. Each response was recorded while a blank screen was visible for 3000 ms (responses longer than this deadline were not included in the analyses). Each participant performed a total of 336 trials; of these, 10% were cue-invalid and the remaining 303 cue-valid trials were randomized and balanced in order to consider the following conditions: spatial relation (CAT, COO, NoCh), cue size (‘large’ and ‘small’) and visual field (left and right). The presentation sequence (Figure 1 A) was controlled by E-Prime 1® software, which also collected the participants’ responses.

### MEG Recordings

The magnetic field was recorded by using a whole head MEG system consisting of 165 SQUID integrated magnetometers and located inside a good quality magnetically shielded room [46]. Evoked magnetic fields were bandpass filtered at 0.16–250 Hz and recorded at 1 kHz sampling rate. To determine the position of the subject head with respect to the sensor, the magnetic field generated by five coils placed on the scalp was recorded before and after measurement session. The location of the coils of four anatomical landmarks on the subject head were digitised by means of a 3D-Digitizer (Polhemus, 3Space Fastrak).

Cardiac and ocular activities were also monitored by means of bipolar electrodes placed on the chest and on peri-orbital region so as to filter out possible heart contaminations on the MEG signals and to exclude from the analysis trials including eye movements from the fixation point. Heart contaminations were filtered out on the MEG raw signals by means of an adaptive algorithm using orthogonal projections [47], [48]. A high-resolution whole head structural image MRI was performed via a Philips scanner at 3 T using 3D T1-TFE sequence. Spherical oil capsules were applied on the anatomical landmarks to allow coregistration of MEG and MRI coordinate systems. Anatomical images were then transformed into stereotaxic coordinates of the Talairach space.

### Data Analysis

**Figure 2:**
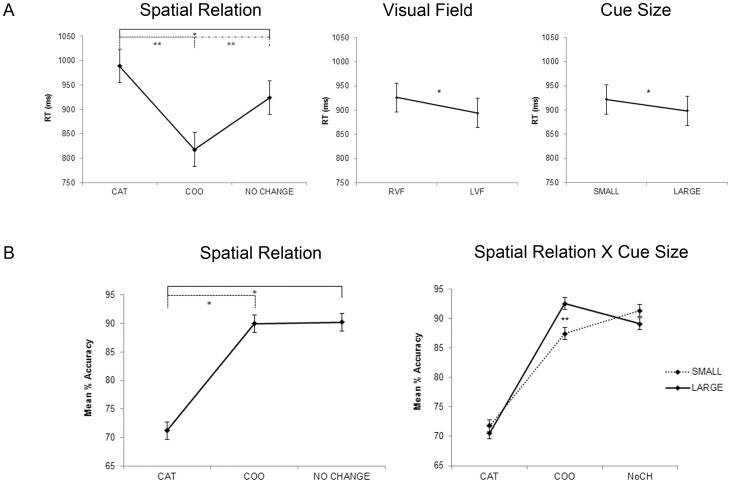
Behavioural results. A: Response time for spatial relation, visual field and cue size main effects; B: Accuracy for spatial relation main effect and interaction between spatial relation and cue size. Error bars represent the standard deviations for the main effect and 95% confidence intervals computed with the formula of Loftus and Masson [72] for within-subject designs.

Cue-invalid trials were not included in the behavioural analysis and they served as filler trials that also signalled a probabilistic relation between a cue and a specific spatial location. Moreover, the low number of cue-invalid trials did not allow performing electrophysiological analysis, since the number of the trial was too small to obtain a good signal-to-noise ratio in the mean evoked magnetic field. For each of the twelve conditions, valid trials with correct responses were averaged over the timeline from 0 ms, corresponding to the matching stimulus onset, until 1000 ms; that is, approximately the maximal response time in which, on average (see Figure 2), the behavioural responses were given. A baseline level for the calculation of the amplitudes of the evoked magnetic fields was set as the mean value of the entire epoch magnetic field (0–1000 ms). The period preceding the match stimuli was not chosen as baseline because it could be contaminated by cortical activations due to the cue presentation or to the attended match stimulus.

In order to identify brain areas involved in the task, mean evoked brain responses were obtained for CAT, COO and NoCh conditions regardless the size of the cue and the visual field of the presentation of the stimuli.

Generators of MEG evoked responses were obtained by means of LORETA analysis (low resolution brain electromagnetic tomography) which is a functional brain imaging method based on the electrophysiological and neuroanatomical constraints [49]. An imaging approach such as LORETA, makes no a priori assumption on the number of sources that can be driven from data and provides a blurred image of a point source centered on the location of maximal activity, even for shallow and correlated sources. Experimental and theoretical investigations have supported the validity of LORETA demonstrating that it has small errors of localization and lower spatial dispersion [50], [51] compared to other inverse solution methods, but it is not well suited for focal source estimation compared to Linear Constrained Minimum Variance beamforming method in the time [52] and frequency domain [53].

Using the BESA software, the activity at each voxel of the 3D volume grid having a spacing of 7 mm was estimated for each 1 ms time point [54]. The entire epoch of 1000 ms was divided into temporal intervals 50 ms long from the onset of the matching stimulus (twenty time intervals). Then the activity of each voxel inside the grid was averaged across each time interval, obtaining activation maps with 50 ms resolution for each condition (CAT, COO and NoCh). Subsequently, the grand average across subjects was performed in order to obtain activation maps over the structural MRI in Talairach space for the CAT, COO and NoCh conditions separately. From these, clusters of activations common to the conditions (CAT, COO and NoCh), as also evidenced in previous PET and fMRI studies on spatial relations [14], [15], were selected from the three activation maps. From each cluster of activation, we selected a region of interest (ROI) including the voxel of maximal activity and the 26 nearest neighbour voxels, as it has been reported in previous studies [55], [56]. On the basis of the hypothesis that the different sources involved in the spatial relation task could activate with different temporal intervals, we determined objectively the time interval of the maximal activity for each source by a data-driven statistical approach. We performed one-way ANOVA analysis for each ROI separately on normalized intensity strength averaged across all conditions (discarding type of spatial relations, cue size and visual field of presentation) with the twenty temporal intervals of 50 ms as main factor, in order to obtain the time intervals in which the source activities were higher than all the rest of the epoch.

Thus a within-subject statistical analysis was performed on the integral of the source strength across the time interval with the statistical maximal activity, determined previously.

For the comparison on the intensity of activations of clusters among all conditions, LORETA analysis was re-performed on mean evoked magnetic fields of the 12 conditions (2 cue size × 2 visual field × 3 spatial relations). Then, the mean activity across each cluster was performed for each condition and subsequently averaged across each 50 ms time interval. Intensity values were normalized to the maximal value obtained for each subject among all conditions in order to eliminate inter-subject variability on source strength.

The intensity of each ROI for each of the twelve conditions was evaluated as the integral of the source strength across the time interval obtained by the one-way ANOVA described previously. In addition, for each of the twelve conditions, the peak latency of the source waveforms with 1 ms resolution was evaluated for each ROI. The peak latencies of the sources were calculated from the onset of the matching stimulus.

Within-subject statistical analyses were performed on the activity strength and peak latency of each cluster, on response accuracy and on response times by means of repeated measures ANOVA with three factors and 12 conditions: cue size (large, small), visual field (left, right), spatial relation (CAT, COO, NoCh). A post-hoc analysis using the Duncan test was used for multiple comparisons. The level of statistical significance was set at 5% (p<0.05).

## Results

### Behavioural Results

The ANOVA on response accuracy (Mean % accuracy) as the dependent variable showed a significant effect of the spatial relation factor (F_2,42_ = 21.81, p<0.001). Participants were less accurate for the CAT than both NoCh and COO (p<0.001) spatial relations, reflecting the greater difficulty of the categorical spatial relation task than the other spatial relation task as it was expected. The spatial relation × cue size interaction was also significant (F_2,42_ = 7.94, p<0.01); participants were less accurate when they had to evaluate COO-small cue than COO-large cue trials (p<0.003).

The ANOVA with RT as the dependent variable showed significant effects of the spatial relation (F_2,42_ = 33.08, p<0.001), visual field (F_1,21_ = 14.31, p<0.01) and cue size (F_1,21_ = 9.57, p<0.01). Participants were faster during COO trials than both CAT and NoCh trials (p<0.0001), and during NoCh trials than CAT trials (p<0.004) confirming the greater difficulty of the CAT with respect to COO and NoCh condition; in addition, they were faster when matching stimuli were presented in the left than in the right visual field (p = 0.001) and when matching stimuli followed the large cue than the small cue (p<0.01).

Figure 2 shows all significant results in response times and accuracy.

### MEG Results

**Figure 3:**
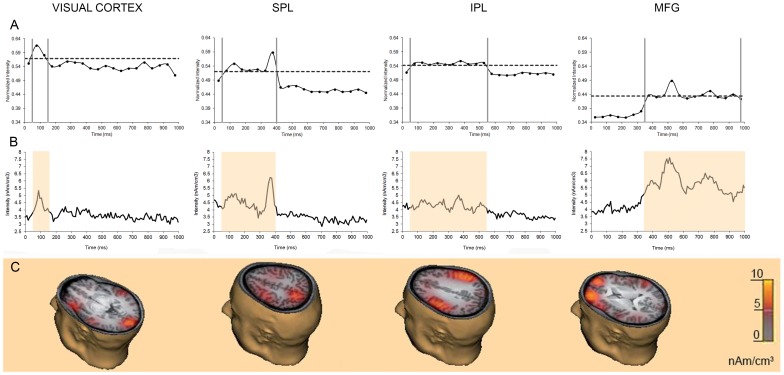
MEG results. A: Mean normalized intensity across all conditions and subjects over time for selected ROIs. Vertical bars indicate the temporal intervals with the highest intensity values established by statistical analysis. Horizontal line indicate the cut-off value (mean+sd). B: Group mean temporal activity was averaged for all conditions for areas showing strongest activity after the matching stimulus. Coloured bars indicate temporal intervals determined previously from statistical analysis and used in the following statistical analyses to compare source activity across conditions (50–150 ms for visual cortex, 50–400 ms for superior parietal lobe, 50–550 ms for inferior parietal lobe and 350–1000 ms for middle frontal gyrus); C: Spatial maps of activations for each areas.

LORETA analysis on spatial relations (CAT, COO and NoCh) showed clusters of activation in bilateral Inferior Parietal Lobe (IPL), bilateral Middle Frontal Gyrus (MFG), medial Superior Parietal Lobe (SPL) and Visual Cortex. Coordinates of the centre of the ROIs in Talairach space are shown in Table 1. These ROIs showed different temporal patterns of activation across time intervals from the onset of the matching stimulus (0 ms) to the maximal response time (1000 ms). ANOVA results on mean intensity strength across all conditions for the 20 time intervals showed a main effect of time for Visual Cortex (F_19,380_ = 4.68, p<10^−6^), SPL (F_19,380_ = 8.93, p<10^−6^), IPL (F_19,380_ = 4.61, p<10^−6^) and MFG (F_19,380_ = 8.31, p<10^−6^). Specifically, Duncan post-hocs showed that, for the Visual Cortex, the activity in the 50–150 ms was greater than the 0–50 ms and the 150–200 ms intervals (0.05<p<10^−5^); for the SPL the activity in the 50–400 ms time interval was greater than for both the 0–50 and 400–1000 ms time intervals (0.05<p<10^−5^); for the IPL the activity in the 50–550 ms time interval was greater than for both the 0–50 and 550–1000 ms time intervals (0.05<p<10^−5^); for MFG the activity in the 350–1000 ms time interval was greater than 0–350 ms time interval (0.05<p<10^−5^). In addition, in the time intervals highlighted by the statistical comparisons, the normalized intensity of the sources was higher than the cut-off value performed as the mean intensity plus one standard deviation across all intensity values on the entire 0–1000 ms epoch (Figure 3 A).

**Table 1:** Involved areas.

Region	BA	*x*	*y*	*z*
Visual Cortex	18	4	−80	4
Superior Parietal Lobe	7	4	−59	45
Left Inferior Parietal Lobe	40	−49	−31	31
Right Inferior Parietal Lobe	40	53	−31	31
Left Middle Frontal Gyrus	10	−31	46	10
Right Middle Frontal Gyrus	10	32	46	10

Brodmann areas (BA) and Talairach coordinates in mm of the center of clusters.

Figure 3 B and C show the temporal profile with 1 ms resolution averaged across subjects and conditions as well as the spatial maps of the cluster of activations for a representative participant. The temporal intervals showing the maximal activity for each area were highlighted with yellow bars.

For each area, the activity strength and the peak latency in the time interval showing the maximal activity was different among conditions. Within-subject statistical analyses performed on the normalized activity strength showed significant main effects and interactions. Interestingly, some main effects were common to all activation clusters. Indeed the main effects of visual field and spatial relations were significant in all involved brain areas. The activity strength was larger when the spatial cue and matching stimulus were presented in the right than the left visual field (see Table 2); a finding that is consistent with the behavioural finding showing that responses to matching stimuli presented to the right visual field were slower than those to the left visual field. The main effect of spatial relation showed that the intensity strength was larger in the CAT condition than in NoCh for all brain areas. In visual cortex and parietal areas the intensity strength in CAT condition was also larger than COO condition. This result is consistent with the present behavioural findings of slower and less accurate responses in CAT trials than COO and NoCh.

**Table 2:** Significant statistical results.

Area	Significant Effects		F	p	Post hoc comparison	p
Visual Cortex						
(50–150 ms)	Visual Field	RVF>LVF	29.29	.000		
						
	Spatial Relations	CAT>COO;CAT>NoCh	6.99	.002		
	Cue × Spatial Relations		10.45	.000	NoCh Large Cue>Small Cue	.000
	Visual Field × Spatial Relations		3.32	.046	CAT (RVF>LVF)	.000
SPL						
(50–400 ms)	Visual Field	RVF>LVF	5.88	.025		
	Spatial Relations	CAT>COO; CAT>NoCh	17.19	.000		
	Cue × Visual Field × Spatial Relations		7.69	.001	CAT RVF (Small Cue>Large Cue)	.037
					COO Large Cue (RVF>LVF)	.008
					NoCh LVF (Large Cue>Small Cue)	.056
IPL						
(50–550 ms)	Visual Field	RVF>LVF	17.32	.000		
	Spatial Relations	CAT>COO; CAT>NoCh	13.04	.000		
	Hemisphere × Visual Field		5.42	.031	Left Hemisphere (RVF>LVF)	.000
	Visual Field × Spatial Relations		7.54	.002	CAT (RVF>LFV)	.000
	Hemisphere × Cue × Spatial Relations		3.69	.034	**Right Hemisphere** **(COO Large>COO Small)**	.031
					(NoCh Large>NoCh Small)	.044
					**Left Hemisphere** **(CAT Small>CAT Large)**	.002
					(NoCh Large>NoCh Small)	.000
	Cue × Visual Field × Spatial Relations		8.05	.001	CAT (Small cue RVF>Small cue LFV)	.000
					COO (Large cue RVF>Large cue LVF)	.007
					NoCh LVF (Large Cue>Small Cue)	.011
MFG						
(350–1000 ms)	Visual Field	RVF>LVF	13.82	.001		
	Spatial Relations	CAT>NoCh	4.46	.018		
	Hemisphere × Visual Field		8.36	.009	Right Hemisphere (RVF>LVF)	.000
					Left Hemisphere (RVF>LVF)	.002
	Visual Field × Spatial Relations		4.49	.017	CAT (RVF>LVF)	.001
					RVF (CAT >COO)	.013
					RVF (CAT >NoCh)	.000
	Cue × Visual Field × Spatial Relations		9.8	.000	COO Large Cue (RVF>LVF)	.013
					COO RVF (Cue Large>Cue Small)	.012
					CAT Small Cue (RVF>LVF)	.000
					NoCh LVF (Large Cue >Small Cue)	.024

Statistical results on the activity strength in the visual cortex, superior parietal lobe (SPL), inferior parietal lobe (IPL) and middle frontal gyrus (MFG) for selected temporal intervals. The most important statistical findings are reported in bold.

Significant interactions were also found in all areas. Specifically, in visual cortex, the significant interaction between cue size and spatial relation as well as between visual field and spatial relation revealed that in NoCh condition the activity strength was higher when the attention cue was large compared to small and, in the CAT condition, the activity strength was higher for the RVF than the LVF, respectively. In the superior parietal lobe the interaction between cue size, visual field and spatial relation was significant, showing that in the CAT condition presented in the RVF the activity was higher for the small than large cues whereas, in the COO condition, the activity was higher following large cues when the stimulus was presented in the RVF than the LVF.

**Figure 4:**
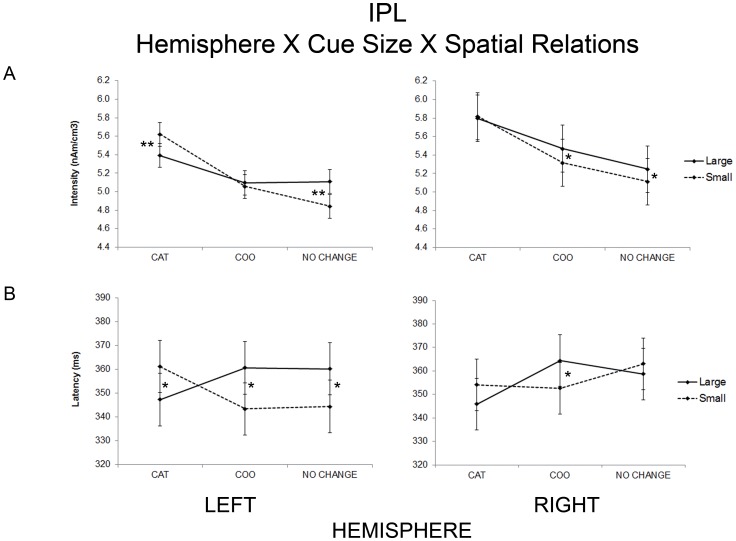
Significant statistical results. Relevant statistical results for the strength (A) and peak latency (B) obtained for the inferior parietal lobe. In the left hemisphere categorical spatial relations yielded greater activity and longer latencies when the attention window was small; in the right hemisphere, coordinate spatial relations yielded greater activity and longer latencies when the attention window was large. Error bars are computed according to the formula for within-subject designs by Loftus and Masson [72] * p<005; **p<0001, significant post hoc comparisons.

In the light of the present theory, the most important findings were observed within the inferior parietal lobe. Crucially, within the IPL the interaction hemisphere × cue × spatial relation was significant and post-hoc comparisons showed that in the right hemisphere, the COO condition yielded higher activity when the attention window was large than small, whereas in the left hemisphere, the CAT condition provided higher activity when the attention window was small than large (Figure 4 A, Table 2).

Interestingly, for the NoCh condition the activation of IPL was greater for a large than for a small attention window in both the left and right hemispheres, suggesting that in NoCh a large attention window may engage a larger neuronal population than a small attention window. In addition, for both the frontal and parietal areas, the activity in NoCh spatial relation was higher for large than small cue when the stimulus was presented in the LVF.

In the middle frontal gyrus, two significant interactions indicated that both for the RMFG and LMFG the intensity was higher in the CAT condition when the stimulus was presented in the RVF than the LVF. The interaction between cue size, visual field and spatial relation indicated that, for the large cue-COO and small cue-CAT conditions, the intensity was higher when the stimulus was presented in the RVF than the LVF.

All statistically significant results for each area in the temporal interval showing the maximal activity are reported in Table 2.

Statistical analysis on peak latency replicated the crucial findings within the inferior parietal lobe as seen above for the intensity strength (see Figure 4 B). Indeed within the IPL the interaction hemisphere × cue × spatial relation was significant and post-hoc comparisons showed that, in the RIPL and in the LIPL, the COO condition yielded longer latency when the attention window was large compared to small, whereas in the LIPL, the CAT condition resulted in longer latency when the attention window was small instead of large. For the NoCh condition the latency of LIPL was longer for large than for small attention windows. In addition the peak latency for the Visual Cortex in the CAT condition was longer when the attention window was large than small; for the superior parietal lobe no interaction was significant whereas in the middle frontal gyrus the interaction hemisphere × visual field × spatial relation reached significance. Specifically, in the RMFG, the CAT condition yielded longer latency when it was presented in the left than in the right visual field, whereas the COO condition yielded longer latency when it was presented in the RVF than the LVF. All significant statistical results on peak latency for each ROI are reported in Table 3.

**Table 3:** Significant statistical results.

Area	Significant effects		F	p	Post hoc comparison	p values
Visual Cortex						
(50–150 ms)	Cue × Spatial Relations		5.50	.008	CAT Large>CAT Small	.030
	Cue × Visual Field × Spatial Relations		5.09	.011	LVF (CAT Large>CAT Small)	.019
					LVF (NoCh Large>NoCh Small)	.050
SPL						
(50–400 ms)	Cue	Large>Small	4.72	.042		
	Spatial Relations	NoCh>CAT;COO	3.42	.043		
IPL						
(50–550 ms)	Cue × Spatial Relations		7.24	.002	CAT (Small Cue>Large Cue)	.041
					COO (Large Cue>Small Cue)	.008
	Hemisphere × Cue × Spatial Relations		3.86	.029	Right Hemisphere(COO Large>COO Small)	.035
					Left Hemisphere(CAT Small>CAT Large)	.011
					(COO Large>COO Small)	.002
					(NoCh Large>NoCh Small)	.004
MFG						
(350–1000 ms)	Hemisphere	LH>RH	4.17	.055		
	Spatial Relations × Visual Field		6.22	.004	CAT (LVF>RVF)	.008
					RVF (COO>CAT)	.041
					LVF (CAT>COO)	.050
					RVF (NoCh>CAT)	.049
	Hemisphere × Spatial Relation × Visual Field		9.32	.000	RH CAT (LVF>RVF)	.000
					RH COO (RVF>LVF)	.002
					RH LVF (CAT>COO)	.000
					CAT LVF (RH>LH)	.033
					CAT RVF (RH>LH)	.003
	Hemisphere × Cue × Spatial Relation × Visual Field		7.13	.002	COO Large LVF (LH>RH)	.050
					CAT Small RVF (LH>RH)	.007
					NoCh Small LVF (LH>RH)	.001
					RH NoCh LVF (Large>Small)	.007

Statistical results on peak latency in the visual cortex, superior parietal lobe (SPL), inferior parietal lobe (IPL) and middle frontal gyrus (MFG) for selected temporal intervals.

## Discussion

The present MEG study identified the parietal lobes (both in their inferior and superior portions) as well as the medial regions of the frontal lobes as playing a key role for spatial relation processing. Although regions of the parietal cortex have been implicated in highly dissimilar tasks, including reorienting attention, episodic memory retrieval, understanding language, performing mental calculations [57], [58], our study aimed at directly investigating the effect of the scope of attention on the neural correlates involved in processing spatial relations. Brain areas involved in the present study were indeed remarkably consistent with those areas shown to be crucial in previous patient studies as well as TMS, divided-visual-fields, or neuroimaging studies that specifically tested categorical versus coordinate spatial processing [6], [10], [11], [14]–[16], [43], [59]. Generally, a dorsal fronto-parietal network may be particularly important in representing the spatial positions and relations of the stimuli being compared.

The analysis on the time courses of neural activity showed that the mean peak latency after the onset of the matching stimulus across conditions was about 90 ms for the visual cortex, 350 ms for the superior and inferior parietal lobe and 700 ms for the middle frontal gyrus. The analysis on the activity strength showed that the stimuli presented in the right visual field elicited stronger activity than the stimuli presented in the left visual field for all clusters of activation (Table 2), indicating an intense activity of the contralateral, left hemisphere’s fronto-parietal network. In the present study, given the spatial resolution of the MEG signals, the medial activation within the visual cortex and the superior parietal lobe did not allow revealing any underlying topographic organization of the visual cortex and superior parietal lobe [60] during spatial relation processing. The observed greater activity in the left hemisphere likely reflects the greater difficulty, as indicated by the behavioural results, of the categorical spatial relations task than the coordinate spatial relations in the present paradigm. Several previous neuroimaging studies have shown that task difficulty modulates the activity of specialized neuronal populations [61], [62]. In contrast, it is likely that the frontal areas begin their processing only after the spatial information has been substantially processed within the parietal areas. Activity in the frontal areas may represent spatial working memory processing or other executive control mechanisms that may assist the conversion of one type of spatial representation into another, possibly for an efficient control of shifts in spatial attention and gaze (e.g., a categorical-to-coordinate conversion subsystem, likely to reside in frontal areas, as hypothesized by Kosslyn [3]). However, working memory effects can also be observed already at the level of the visual areas, as shown in van der Ham et al.’s study [25], by the differential patterns of dispersion of activation in retinotopic areas during the interval between the sample and the match stimuli. Interestingly, in the present study, we observed significant activation in the visual areas between 50–150 ms after the appearance of the match and partially overlapping those in the inferior parietal lobes (i.e., 50–550 ms).

The most important result of the present study evidenced that large spatial cues yielded greater activations and longer latencies in the IPL of the right hemisphere for coordinate trials, whereas small cues yielded greater activations in the IPL of the left hemisphere for categorical trials. According to a recent theory of the role of spatial attention in spatial cognition [22], [23], [24], [34], we hypothesized differing modulatory effects of the scope of attention in a categorical versus coordinate spatial task. Previous studies on patients [6] or healthy subjects [5] had investigated only the role of the left and right dorsal system on different types of spatial relations. The parietal lobes of unilateral stroke patients were found to play a crucial role in representing the spatial relations in a complementary manner [6], and another recent fMRI study [63] specifically investigated the encoding of categorical versus coordinate spatial relations during an active navigation task and found strong activations within the parietal cortex of the left hemisphere for the categorical condition.

The present study also led to several counterintuitive findings. First of all, despite both tasks were of a spatial nature, we found preponderant brain activations when the stimuli were presented in the right visual field for both tasks (see Table 2). In the light of the behavioural results, showing slower correct responses when matching stimuli were presented in the RVF than LVF, we surmise that the greater difficulty of the categorical spatial relation task resulted in a strong activation of the left hemisphere, which in turn could have prioritized the processing of contralateral stimuli (i.e., the right visual field’s presentations). Thus, an alternative hypothesis that the difficulty and/or the engagement of the right hemisphere’s attentional mechanisms (e.g., shifting the attention window) can explain hemispheric differences in these spatial tasks [44] is inconsistent with the present results, because in the present paradigm the more difficult task, i.e., the categorical task, resulted in greater activity in the left than the right hemisphere. Note also that, within the present theory [22], it is actually the categorical task that is more likely to engage visuo-spatial shifts of attention (i.e., selecting a narrow focus of attention and, consequently, yielding multiple shifts of attention on separate objects; see also [64]).

An intriguing result was that the side of presentation of the matching stimuli enhanced the effects of the spatial cues in a manner consistent with the theorized advantages (i.e., small cues for CAT trials and large cues for COO trials). Namely, not only small cues resulted in greater activity during categorical trials in SPL, IPL, MFG, but also large cues in the RVF yielded greater activity in these same areas during coordinate trials. These results are highly counter-intuitive but, as such, are not inconsistent with the current theory about the two hemispheres’ specializations for both spatial relation processing and attentional processing, which posits them to be relative and not absolute [65]–[70]. In other words, each hemisphere has the ability to encode and judge both spatial relations or to narrow or expand the focus of attention but each of them can do so with different degrees of proficiency. The present findings show that the attentional benefit of the lateralized cue sizes may be visible for each spatial relation and especially so for the preponderantly activated (left) hemisphere.

However, a boost of activity after RVF presentations was also observed in the ipsilateral, right-sided, medial frontal gyrus. This is the only effect that seemingly contradicts the standard neuroimaging evidence that high-level visual areas have a “preference” for contralateral stimuli; for example, fMRI studies show that not only the primary visual cortex but also the fusiform gyrus in each hemisphere is more activated by face stimuli presented within the contralateral visual hemifield [71]. Again, due to the greater engagement of the left hemisphere in this study, it is possible that all right-sided input was more robustly represented and that such an advantage could reveal itself even within ipsilateral pathways of the processing network.

To conclude, the present findings support the existence of a neural architecture for spatial relation processing that is relatively specialized and depends for its optimal functioning on a strategic use of the focus of attention. We did find neural evidence with the use of MEG supporting the hypothesis that narrowing attention to encompass an area that includes only one of the objects benefits categorical spatial relations, whereas spreading the attention window to encompass an area that includes two objects promotes coordinate spatial relations. In addition, we found that stimuli contralateral to the most activated hemisphere revealed, more robustly than ipsilateral stimuli, the expected modulatory effects of the scope of attention on each type of spatial relation judgment.
